# Combination of Modified Atmosphere and Irradiation for the Phytosanitary Disinfestation of *Trogoderma granarium* Everts (Coleoptera: Dermestidae)

**DOI:** 10.3390/insects12050442

**Published:** 2021-05-12

**Authors:** Qing-Ying Zhao, Tian-Xiu Li, Zi-Jiao Song, Tao Sun, Bo Liu, Xin Han, Zhi-Hong Li, Guo-Ping Zhan

**Affiliations:** 1 MOA Key Laboratory of Pest Monitoring and Green Management, Department of Plant Biosecurity, College of Plant Protection, China Agricultural University, Beijing 100193, China; zhaoqy2021@126.com (Q.-Y.Z.); songzijiao0505@163.com (Z.-J.S.); 13100808152@163.com (T.S.); SY20203192943@cau.edu.cn (X.H.); 2 Institute of Equipment Technology, Chinese Academy of Inspection and Quarantine, Beijing 100123, China; ltx219@126.com (T.-X.L.); liubobj@126.com (B.L.)

**Keywords:** *Trogoderma granarium*, khapra beetle, modified atmosphere, irradiation, combined treatment, synergistic effects, synergism

## Abstract

**Simple Summary:**

The khapra beetle is defined as one of the most important quarantine pests globally, and fumigating by methyl bromide, one of the ozone-depleting substances under the Montreal Protocol, is a routine measure used for phytosanitary treatment. To protect the Ozone layer, an environmentally friendly measure is needed to be developed. The middle- to late-stage larvae and adults were treated with irradiation, modified atmosphere (MA) alone, and their combinations at room temperature of 24–26 ℃. As a result, late-stage larvae are determined as the most tolerant stage. Ionizing radiation was used to enhance the effects of 1% and 2% O_2_ MA treatments that the obvious synergistic effects are presented in all combinations, resulted in saving as high as 60% of the estimated exposure times comparing with MA treatment alone. A total of 111,366 late-stage larvae were exposed to a 1% O_2_ atmosphere for 14 or 15 days after a 200 Gy irradiation, resulted in no survivor in the validating tests. Therefore, the MA-irradiation combination treatment can provide quarantine security at a very high level, it may be combined with international transportation (train or sea container) to disinfest the commodities infested by khapra beetle and other stored products insect pests.

**Abstract:**

The khapra beetle, *Trogoderma granarium* Everts, is defined as one of the most important quarantine pests globally, and fumigation with methyl bromide, an ozone-depleting substance, is a common phytosanitary measure currently used. The modified atmosphere (MA), irradiation, and their combination treatments of *T*. *granarium* larvae and adults were performed at room temperature (24–26 ℃) to develop an ecofriendly phytosanitary disinfestation measure and to shorten the exposure time and overcome treatment disadvantages of irradiation. Late-stage larvae are determined as the most tolerant stage resulted in large LT_99.9968_ values of 32.6 (29.2–37.5) and 38.0 (35.1–41.7) days treated under 1% and 2% O_2_ (with N_2_ balance) atmosphere, respectively. Ionizing radiation was used to enhance the effect of MA and the mortality was highly significantly affected by all the interaction effects, indicating that the synergistic effects present in all the combined treatments. The synergistic ratios, which is defined as the estimated lethal time for MA treatment (LD_90_, LD_99_, and LD_99.9968_), divided by that of combined treatment, were between 1.47 and 2.47. In the confirmatory tests, no individuals recovered from a sum of 111,366 late-stage larvae treated under 1% O_2_ atmosphere for 14- or 15-d after 200 Gy irradiation, which resulted in validating the probit estimations and achieving an efficacy of 99.9973% mortality at 95% confidence level. Therefore, these treatment schedules are recommended to disinfest *T*. *granarium* infecting commodities for phytosanitary purposes under the warehouse, MA packaging, or in combination with international transportation by train or sea container.

## 1. Introduction

The khapra beetle, *Trogoderma granarium* Everts (Coleoptera: Dermestidae), is endemic to India, but viable populations may survive in almost any country in a closed storage environment [1]. The larvae can cause heavy economic losses to stored grains and other food commodities. Damage can be severe with weight losses of between 5–30% and, in extreme cases, 73% worldwide [2]. It is ranked as one of the 100 worst invasive species worldwide [3]. Like most stored product insects, *T*. *granarium* was introduced to other continents in recent centuries through international trade, even though adults do not fly. The khapra beetle is currently present in more than 40 countries of Asia, the Middle East, Africa, and Europe. It is listed as a quarantine species by the European and Mediterranean Plant Protection Organization (EPPO), China, the USA, and other countries [4,5,6]. The beetles have been intercepted many times in the port of Australia, China, the USA, and other countries. Furthermore, the number of interceptions increased steadily in recent decades [4,7,8]. As a result, phytosanitary measures, such as phytosanitary treatments, should be taken for the infested commodities. Therefore, it is necessary to develop the disinfestation measures for the phytosanitary treatment of *T. granarium* and its infesting commodities.

At present, phytosanitary treatment of *T*. *granarium* and its infested commodities include fumigation (methyl bromide, phosphine) and temperature treatment [5,9,10]. Even if the methyl bromide has been defined as the ozone-depleting substance under the Montreal Protocol and should be banned and replaced [11], it was commonly used since the khapra beetle is highly resistant to pesticides, phosphine fumigation, extreme low and high temperatures [3,12,13,14]. In order to protect the ozone layer, environmentally friendly phytosanitary treatment measures including; ionizing radiation, modified atmosphere (MA), and low-pressure treatment have been carried out to demonstrate the potential alternative modalities [5,15,16]. The application of MA has been used for controlling the stored arthropod pests by altering the concentration of oxygen (with N_2_ balance), carbon dioxide, or their combinations in the storage environment of products; an international standard for its phytosanitary uses (ISPM No.44: Requirements for the use of modified atmosphere treatments as phytosanitary measures) have just been approved by the International Plant Protection Convention (IPPC) [16,17,18]. Several studies with MA have been performed, and the results showed that mature (late-stage) larvae of *T*. *granarium* are the most tolerant stage to high CO_2_ and low-oxygen atmosphere [3,5]. Thus far, there are no treatment schedules formulated for phytosanitary disinfestation. Ionizing radiation at a low dose has been used to prevent the development and reproduction of arthropod pests, a minimum absorbed dose of 200 Gy is required for preventing reproduction (failure of F_1_ egg-hatch) of the khapra beetle adult, the most radiation-resistant stage [19,20]. However, both MA and irradiation treatment involves a decrease in aerobic metabolism in insects; they are slow-acting control methods that need longer exposure times [21,22,23,24]. For example, Zhang found that the minimum times leading 100% mortality of *T*. *granarium* mature larvae at 32 ℃ were 52, 27.5, and 13-d irradiated at the dose of 440, 880, 1320 Gy, respectively [25]. Furthermore, late-stage larvae of *T*. *granarium* are stimulated into facultative diapause by unfavorable conditions, including extreme temperatures, humidity, food, and crowded environments [5,26,27]. Diapausing larvae were highly resistant to dry, cold, heat, and hunger, in addition, it was markedly more tolerant to low oxygen tension than non-diapausing larvae [3,18,24].

The additive or synergistic effects of combining two or more disinfestation modalities, for example, irradiation-cold storage combination treatment of Melon fly, *Zeugodacus cucurbitae* Coquillet and Mediterranean fruit fly, *Ceratitis capitata* Wiedemann, and an MA-irradiation combined treatment of the confused beetle, *Tribolium confusum* du Val., have been performed to develop a disinfestation measure, in which the irradiation enhances the effect of MA, but its effects are improved by cold storage [28,29]. Moreover, the combination of MA with vapor heat treatment have also been effectively used to lower down treatment temperature and shorten treatment time for the disinfestation of the codling moth, *Cydia pomonella* L. and the oriental fruit moth, *Grapholita molesta* Busck in apple, peaches, and nectarines [30]; the effects of heat treatment are enhanced by the presence of MA; after that, the treatment schedules based on Heat-MA combinations have already been adopted by the USDA [31] and recommend to the IPPC to formulate an international standard, an annex to ISPM 28 (Draft PT: Vapour heat–modified atmosphere treatment for *C. pomonella* and *G. molesta* on *Malus pumila* and *Prunus persica* (2017-037 and 2017-038)) [16,32]. Therefore, a combination of MA with other insect disinfestation measures, including temperature (especially heat treatment), irradiation, and chemicals, is a feasible means to fulfill the requirements for phytosanitary treatment [3,5,33].

In this research, the MA-irradiation combination treatment was thereby conducted for achieving a high level of mortality (i.e., probit 9 mortality) of *T*. *granarium* in a shorter treatment time to stop further damage to its host commodities, so as to determine the additive or synergistic effects of the combined treatment, and to develop a chemical-free and environmentally friendly phytosanitary treatment schedules alternative to methyl bromide fumigation. Therefore, the adult and middle- to late-stage larvae of *T*. *granarium*, which is respectively determined the most tolerant stage to irradiation and MA [5,34,35], were treated with a low-oxygen atmosphere (1%, 2% O_2_ with N_2_ balance), ionizing radiation alone or their combinations in the following tests: (i). Testing the combined/synergistic effects and examining tolerance to each treatment; (ii) dose-response tests on a single MA and combination treatment, and (iii) confirmatory tests on tens of thousands of the most tolerant stage(s) for validating the probit analysis and confirming the probit 9 treatment efficacy.

## 2. Materials and Methods

### 2.1. Insect Rearing

The khapra beetle progeny used in this study was originally from an intercepted sample which was found in an Iranian commercial ship in 2013 at the port of Suzhou, China; after then, it was reared for generations with pesticide-free groundnut cakes and peanut pieces together in the closed glass bottles. A constant temperature and humidity chamber (Chongqing Weir Experimental Equipment Co., Ltd., Chongqing, China) was used to place the rearing bottles by keeping the condition at 35 ± 1 ℃ and 65 ± 10% R.H. in continual darkness. The adults (newly emerged) and larvae (middle-stage, late-stage, and their mixed stages), which were picked out of the rearing bottles with a fine brush and then placed in a plastic cup (6 cm in diameter and 5 cm in height, ~120 individuals in each cup were used as a treatment), were respectively subjected to treatments and then reared at room temperature (24–26 ℃) in the Key Laboratory of Phytosanitary Treatment, Chinese Academy of Inspection and Quarantine, Beijing, China. During the experiments, strict biosecurity measures have been taken to prevent the khapra beetle from escaping and spreading.

### 2.2. Experimental Design

A conclusion has been reached by Hallman et al. [35] based on analyzing lots of researches that the most developed stage in insect is invariably the most radiation-tolerant when a common measure of efficacy is used. Therefore, the khapra beetle adults which is the most developmental stage should be more tolerant to radiation than others. However, mortality is rarely used for efficacy evaluation in the phytosanitary irradiation treatment [22,36]. For MA-irradiation treatment, irradiation was used to enhance the effects of MA treatment, and then mortality should act as the efficacy criterion. Thus, radiation tolerance to mortality in stages should be compared firstly in the testing.

**Gamma radiation of adults and mixed-stage larvae.** The recommended doses for the hygienic treatment of pulses and cereals are 200, 400–600 Gy, respectively, according to the requirements of the Chinese national hygienic standard (GB14891.8-1997: Hygienic standard for irradiated beans, grains, and their products). To compare the radiation tolerance to mortality, newly emergence adults and mixed-stage larvae (middle to late-stage) of *T*. *granarium* were exposed to gamma radiation at the dose of 200, 400, and 600 Gy, respectively. Each of the doses was replicated three times, and the mortality was checked on 7, 14, 21, and 28-d after treatment. 

**Gamma radiation combination with MA treatments of adults and mixed-stage larvae.** In order to compare tolerance to MA-irradiation combined treatment, the adults and mixed-stage larvae were treated under 1% O_2_ atmosphere for the exposure time of 7, 14, and 21-d, respectively, after gamma irradiation at the dose of 200, 400, and 600 Gy. Each of the time-dose combinations was replicated three times. However, results indicated that all the treated beetles died between days 7–14; therefore, shorter exposure times and intervals should be tested in the following testing.

**MA in combination with X-ray radiation treatments of adults, middle-, and late-stage larvae.** The adults, middle- and late-stage larvae were firstly treated with X-rays at the same dose of 200, 400, and 600 Gy, respectively, and then subject to 1% O_2_ atmosphere treatment with the exposure time of 3, 6, and 9-d, respectively. Each of the dose-time combinations was replicated three times.

**Dose-response test of MA alone or in combination with X-ray treatment of late-stage larvae.** To estimate the lethal time of LT_90_ (the minimum lethal time leading to 90% mortality at a specific confidence level (i.e., 90%, 95%, 99%, where 95% confidence level was used for all the estimations in this research), LT_99_ and LT_99.9968_ of the khapra beetle, middle- and late-stage larvae were respectively subjected to 1% or 2% O_2_ MA treatment alone or in combination with 200 Gy X-ray radiation. The experimental design and exposure times for the dose-response tests are listed in Table 1, where the insects without any treatment were used as control, and each of the exposure times was replicated four times.

**Confirmatory tests.** To validate the estimated minimum time for probit 9 mortality of *T*. *granarium* late-stage larvae, a preliminary validating test was first conducted to determine efficient exposure times used in the following tests. A total of 30,000 (each of 10,000, counted before testing) late-stage larvae were irradiated at 200 Gy, then exposed to 1% O_2_ MA treatment for 13-, 14-, and 15-d, respectively. After then, the exposure time of 15-d was used to perform the remaining confirmatory testing.

### 2.3. Treatments

**Gamma radiations.** All the gamma radiations were completed at the National Institute of Metrology Research Irradiator, Beijing, China, where the primary 1.5 × 10^15^ Bq Cobalt-60 source was used for conducting research. Irradiation reference standard and routine dosimetry were done with the Fricke system [37]. The plastic boxes containing khapra beetle samples were placed 50 cm far away from the center of the radiation source and rotated 180° at mid-exposure. The dose rate measured in the first and second treatments were 8.4 and 8.0 Gy/min, respectively, with the dose uniformity of 1.15 and 1.13, respectively.

**X-ray radiations.** An RS-2000 Pro X-ray irradiator (Rad Source Technologies, Inc., Atlanta, GA, USA) was used to conduct all the X-ray irradiations by using the operating parameters of 220 KeV and 17.6 mA. Every ~120 adults or larvae (middle- or late-stage instars) in a plastic box were irradiated at doses of 200, 400, and 600 Gy, respectively. For the confirmatory tests, late-stage larvae (11,020–25,374, counted during mortality-evaluation) were wrapped up in a plastic bag for irradiation at 200 Gy. The dose rate monitored in all these irradiations was 9.0 Gy/min. 

**MA (Low-oxygen atmosphere) treatment.** All the MA treatments were conducted in the four-liter gastight airbags (Dalian Delin Gas Packaging Co., Ltd, Dalian, China). For each treatment, three or four plastic boxes containing the insect samples (irradiated or none) were placed into one gastight airbag through the opening (at the bottom), followed by the sealing of the airbag, exhausting all the air with a diaphragm pump, and injecting 1% or 2% O_2_ (with N_2_ balance) (Beijing Green Oxygen Tiangang Technology Development Co., Ltd, Beijing, China) into the airbag and kept it for a few minutes [38]. The exhausting-injecting procedure was repeated at least three times to purify the gas in the airbag. Then, all the airbags were placed in one room with a temperature of 24–26 ℃; the gases in the airbags were refined every two days until the exposure times reached.

### 2.4. Insect Rearing after Treatments

The treated cups or boxes were taken out of the airbags and kept for another seven days at room temperature. Then, the number of larvae, pupae, and adults (dead or survivor) were counted. Mortality was evaluated based on non-movement with acupuncture and/or color changes of the insect body.

### 2.5. Data Analyses

Mortality data for irradiation, MA treatment alone or their combinations were corrected by using Abbott’s formula [39] and then subjected to two-way or three-way Analysis of Variance (ANOVA) to analyze the individual effects of main factors and their interaction effects; means (±SD, for all the mortality) were compared by Tukey’s multiple comparison tests; where DPS software was used in the analysis [40]. The dose–response (time-mortality) data on MA treatment alone or in combination with irradiation were analyzed with Probit model by using PoloPlus 2.0 program to estimate the lethal exposure time (using non-transformed exposure times), in which any mortality data between 0 and 100%, and the shortest exposure time causing 100% mortality were used in the analysis [38,41]. Pare-wise comparison tests were performed by calculating the 95% confidence limits (CIs) of the lethal dose ratios at LT_90_, LT_99_, and LT_99.9968_ so as to compare the significance of the tolerance of the khapra beetle between larval stages and treatments at different O_2_ levels. If the 95% CIs excludes 1, then the LTx values are significantly different [38,42,43]. 

To determine the additive or synergistic effects in combined treatments, the synergistic ratios (SRs), which are defined by Hewlett and Plackett [44] and have been used by Chadwick [45], who call it the factor of synergism, and Lee et al. [46] in the combination of two pesticides or fumigants, was also calculated from the Equation (1).

(1) $$\mathrm{SRs}=\frac{\mathrm{LTx}\;\mathrm{of}\;\mathrm{MA}\;\mathrm{alone}}{\mathrm{LTx}\;\mathrm{of}\;\mathrm{MA}\;+\;\mathrm{Irradiation}\;\left(\mathrm{combined}\;\mathrm{treatment}\right)}$$

where: LTx presents the estimated lethal time, for example LT_90_:

SRs = 1 describes additive action,

SRs < 1 describes antagonism,

SRs > 1 describes synergism.

For the confirmatory tests, the mortality proportion (1 − *Pu*) associated with treating a number of khapra beetle with zero survivors is given by Equation (2) for a defined confidence level.

1 − *Pu* = (1 − *C*)^1/*n*^ (2)

where *Pu* is the maximum allowable infestation proportion, *C* is confidence level, and *n* is the number of test insects. Furthermore, the number (*n*) treated in confirmatory tests should be adjusted based on control survivorship [47,48,49].

## 3. Results

### 3.1. Effects of Gamma Radiation

**Figure 1:**
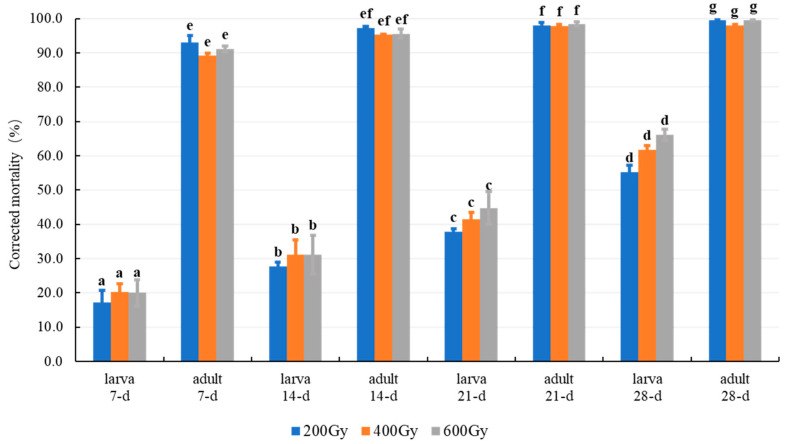
Percentage mortality of *T. granarium* adult and larvae treated with 200, 400, and 600 Gy gamma radiation for the duration of 7, 14, 21, and 28-d at 24–26 ℃. Means (±SD) followed with different letters are significantly different (*p* ˂ 0.05; Tukey test).

Mortality of *T*. *granarium* generally increased with increasing exposure time (from 7 to 28-d) and radiation dose (from 200 to 600 Gy), while complete mortality was not achieved in adults or mixed-stage larvae either (Figure 1). The differences of corrected mortality were significant for the main factors of stages (*F*_1,71_ = 2505.22, *p* ≤ 0.0001) and times (*F*_3,71_ = 82.08, *p* ≤ 0.0001), and for the two-way interaction effects of stage × time (*F*_3,48_ = 40.83, *p* ≤ 0.0001) and stage × dose (*F*_2,48_ = 3.37, *p* = 0.0427); therefore, mortality for both larvae and adult increased significantly with increasing time, and the mean mortality value (±SD) for adults (96.0 ± 3.56%) was significantly larger than that of larvae (37.9 ± 16.91%), indicating that the larvae are significantly more tolerant to irradiation than adults (mortality was used for evaluating treatment efficacy). However, the main effects of radiation dose (three-way: *F_1_*_,71_ = 1.67, *p* = 0.1995; two-way for larvae: *F*_2,35_ = 2.47, *p* = 0.1060; 2-way for adults: *F*_2,35_ = 3.23, *p* = 0.0573) and the interaction effects of dose by stage and/or time (two-way for larvae: *F*_6,24_ = 0.24, *p* = 0.9586; two-way for adults: *F*_6,24_ = 0.64, *p* = 0.7011) were insignificant, indicating that there are no synergistic effects between dose and times after irradiation. As a result, mortality of *T*. *granarium* increased slowly with increasing dose and there is no significant difference among gamma radiation at 200, 400, and 600 Gy.

### 3.2. Effects of Combined Treatments

#### 3.2.1. Effect of MA in Combination with Gamma Radiation

**Figure 2:**
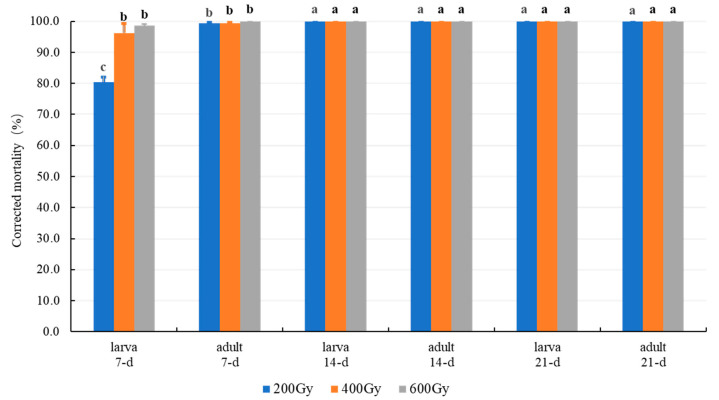
Percentage mortality of *T. granarium* adult and mixed-stage larvae treated at 24–26℃under 1% O_2_ atmosphere for the exposure time of 7, 14, and 21-d after 200, 400, and 600 Gy gamma radiation. Means (±SD) followed by different letters are significantly different (*p* ˂ 0.05; Tukey test).

Most of the adult and mixed-stage larvae of *T*. *granarium* died within 7-d, and all of them died within 14-d when they were exposed to 1% MA treatment after gamma radiation (Figure 2). Results of three-way ANOVA showed that the difference in mortality was highly significant (*p* ≤ 0.0001) for the main factors of the stage (adult ˃ larvae), dose (600-Gy ≈ 400-Gy ˃ 200-Gy), and exposure time (21-d = 14-d ˃ 7-d), and for all the interaction effects. Therefore, larvae are also more tolerant to MA-irradiation combined treatment than an adult, just as irradiation treatment alone (Figure 1). 

For the two-way ANOVA, the interaction effects of dose × exposure time and main effects of dose were significant for the mixed-stage larvae (interaction *F*_4,18_ = 15.26, *p* ≤ 0.0001; dose: *F*_2,26_ = 15.26, *p* ≤ 0.0001), but they were insignificant for adults (interaction *F*_4,18_ = 2.00, *p* = 0.1378; dose: *F*_2,26_ = 2.00, *p* = 0.1639), resulted in the larval mortality for 200-Gy + 1%O_2_ (93.5 ± 10.3%) is significantly less than that of 400-Gy + 1%O_2_ (98.7 ± 2.6%) and 600-Gy + 1%O_2_ (99.5 ± 0.8%), and there is not any significant difference among 200, 400, and 600 Gy irradiation-MA combined treatment for the adults. This mysterious result may be due to the long exposure time that results in a very high level of mean mortality (larvae: ≥80.5%; adult: ≥99.4%) (Figure 2). Then, a shorter exposure time and intervals should be tested to determine the interaction and main effects of radiation dose.

#### 3.2.2. Effect of Combination MA with X-ray Radiation

For MA and X-ray combination treatment of *T*. *granarium*, results derived from three- and two-way ANOVA showed that the effects were highly significant for all the main factors and their interactions (*p* ≤ 0.0001). The mortality within a stage increased significantly with increasing radiation doses and exposure times; the least mortality for late-stage larvae (77.4 ± 20.6%) means it is the most tolerant stage, followed by middle-stage larvae (87.3 ± 17.4%), while the adult is the least radio-tolerant stage with the largest mortality of 93.5 ± 8.4% (Table 2).

In comparison with the new emergence adults, larvae (middle- to late-stage) have been determined more tolerant to gamma radiation alone (Figure 1) or in combination with a low-oxygen atmosphere in the previous treatments (Figure 2), while late-stage larvae are more tolerance to combined treatment than middle-stage larvae (Table 2), therefore, late-stage larvae are the most tolerant stage that should be used to conduct the dose-response and confirmatory testing. Furthermore, the largest mortalities were obtained in the combinations of 600Gy-9d and 400Gy-9d, followed by 600Gy-6d and 200Gy-9d for the treatment of late- and middle-stage larvae (under 1% O_2_ atmosphere), suggesting that there are four kinds of optimal combinations that can be used for the controlling strategies. Because the effects of the radiation dose were insignificant (Figure 1) and irradiation is costly comparing with MA treatment, the lowest dose of 200 Gy that can provide quarantine security at the level of probit 9 is the optimum dose to be used in the combination treatment [19,20].

The outcomes of a two-factor analysis are quite complex, during a two-way ANOVA, the main effects are not necessary to be interpreted if the interaction effects are significant [40]. All the interaction effects of dose × time and the main effects of dose were highly significant for the MA-irradiation combination treatments (Table 2), but they are insignificant for gamma radiation alone (Figure 1), indicating that distinguished synergistic effects present in all the MA-irradiation combined treatments, and the main effects of radiation are dominated by the interaction effects of dose × time. This is also the possible reason for the large SRs value that has been obtained in all four kinds of combination treatments (Table 3 and Table 4).

### 3.3. Estimating Lethal Times

Parameters of the probit analysis for middle- and late-stage larvae of *T*. *granarium* treated under 1% or 2% O_2_ atmosphere alone or in combination with 200 Gy X-ray irradiation are presented in Table 3. The smaller value of heterogeneity (chi-square divided by degrees of freedom) means a good fit to the data, and lacking 100% mortality data in the dose-response tests may lead to an unsatisfactory estimation; therefore, good estimation had achieved in all treatments, except for the late-stage larvae treated under 1% (mortality ≤ 94.6 ± 4.7%) or 2% O_2_ MA-irradiation combination that they have larger 95% confidence intervals (CIs). 

For middle-stage larvae of *T*. *granarium*, the positive slope in all treatments was larger than that for late-stage instars, as a result, the estimated mean values were less than that for late-stage instars; in addition, both of the lethal dose ratios test and 95% CIs overlap tests indicated that the difference is significant (Table 3); therefore, late-stage larvae are significantly resistant to MA alone or combination treatment than the middle-stage instars; furthermore, to reduce further damage, the shortest exposure times of 13.2-d (11.9–15.1) which leading the probit 9 mortality of late-stage larvae under 1% O_2_ atmosphere (Table 3) should be used to conduct the following confirmatory tests.

### 3.4. Synergistic Ratios

Equation (1) was used to calculate the synergistic ratios (SRs, Table 4) based on the estimated mean value of lethal times in Table 3. The value of SRs based on LT_90_, LT_99_, and the extrapolated LT_99.9968_ were very closed, ranging from 1.47 to 2.47; suggesting that the combination of MA and irradiation have presented obvious synergistic effects, which may save about 32 to 60% of the exposure times comparing with MA treatment alone. In addition, greater synergistic effects have been achieved for late instars comparing with middle-stage larvae; likewise, more efficient treatments have been achieved under 1% O_2_ comparing with 2% O_2_ atmosphere. Therefore, late-stage larvae treated under 1% O_2_ MA-irradiation combination, which has been determined as an optimal combination (Table 2), and obtained the largest SRs mean (±SD) value of 2.43 ± 0.05 (Table 3), is the most optimal combination. 

### 3.5. Confirmatory Tests

The exposure times of 13, 14, and 15-d, which was estimated by the probit model (Table 3), were used for the preliminary validation tests; however, 1 survivor was found in the treatment of 13-d exposure (Table 5). After that, only 15 days of exposure time were performed in the remaining confirmatory tests. As a result, no survivors were found in a total of 901,366 treated late-instar larvae. Thus, the treatment efficacy (1-*Pu*) calculated from formula 2 is 99.9973% (counting the 20,000 larvae treated in the preliminary validating tests), assuming the confidence level at 95%, the estimation derived from the probit model was thereby validated. In addition, the number treated in the confirmatory tests should be adjusted to account for the percentage of survival in the control (96.8–98.1%), then, the adjusted number is 108,621, and the efficacy is 99.9970% at a 95% confidence level.

## 4. Discussion

Ionizing radiation and MA treatment are currently used measures for disinfecting and disinfestation of quarantine arthropod pests or microorganisms, both of which are environmentally-friendly but slow-acting measures; irradiated but living insect may be another obstacle to be overcome for the application of phytosanitary irradiation treatment [15,22,23,33]. The present results indicated that the minimum exposure times for probit 9 mortality of *T*. *granarium* late-stage larvae were 32.6 (29.2–37.5) and 38.0 (35.1–41.7) days (Table 3) under in 1% and 2% O_2_ atmosphere at room temperature, and more than four weeks are need for complete mortality when the beetle irradiated at the dose of 200 to 600 Gy (Figure 1). However, obvious synergistic effects of their combinations have been demonstrated in the present (Table 3, Table 4 and Table 5) and other studies to be used for preservation treatments and insect disinfestation (i.e., *T*. *confusum*) to improve effectiveness, and to reduce costs, treatment time, and product damage [28,33,50]. For a combination of irradiation with other treatments, the desired response (efficacy evaluation) should be determined firstly since irradiation is different from any other treatment measures, and then the most tolerant stage(s) and additive/synergistic effects should be investigated and confirmed afterward [29,33]. The desired response for MA against stored-product insects is achieving mortality, whereas the desired response for irradiation is typically the prevention of adult emergence or adult sterility [15,22]. For the MA-irradiation combination treatment, there are two choices: the use of MA to modify the response to irradiation, such as Follett and Snook [29] choose cold storage to modify the response to irradiation treatment of two kinds of fruits flies; or as the use of irradiation to modified response to MA. For the present combined phytosanitary treatment of *T*. *granarium*, we chose the former to measure mortality for efficacy evaluation; the advantages for this selection are conducive to overcoming the major obstructs that affects the application of phytosanitary irradiation by the presence of living insects and preventing further damage to the stored products and foodstuff [22,33,48,51]. 

Generally, irradiation with gamma rays or X-rays have the same effects on insects; and the most developed adult should be the most tolerant stage since the radiation tolerance increases with their developmental stage by using a common criterion for efficacy evaluation, for example preventing developments or reproduces of adults [22,33,35]. However, when mortality is used for treatment efficacy criteria, the tolerance sequence of *T*. *granarium* has been changed. Zhang [26] found that the mature larvae are more resistant to gamma radiation than pupae and adult treated at 32 ℃ with the radiation dose of 440, 880, and 1320 Gy, respectively; similarly, in the present research, middle- to late-stage larvae are determined to be more tolerant than adults in the gamma radiation alone or combined with MA treatment (Table 2 and Table 3; Figure 1 and Figure 2). The reason adults are more sensitive to radiation (causing mortality) than late-stage larvae, is possibly due to the slow-acting effects of radiation, the short life span (female: 14–15-d; male: 15–19-d, at 25 °C), and feeding habits (the adult rarely eat or drink) of the khapra beetle [1,22,52]. 

For the combined treatment of late-stage larvae *T*. *granarium*, previous results have shown that it is the most tolerant stage to low-oxygen or high CO_2_ MA treatment, especially the diapausing larvae [1,3,26]. Fortunately, late-stage larvae were also determined more tolerance to low-oxygen MA alone or in combination with irradiation than middle-stage larvae (including adults to the combinations) in our testing (Table 2 and Table 3). Consequently, late-stage larvae were used to conduct the following dose-response tests of low-oxygen MA alone and in combination with irradiation. Finally, the estimated LT_99.9968_ of 13.2-d (11.9–15.1) for late-stage larvae was validated by treating a total of 111,366 late-stage larvae (Table 4 and Table 5), resulted in high treatment efficacy of 99.9973% or 99.9970% (correcting with control mortality) at 95% confidence level [47,48,49,50]. This treatment efficacy may fulfill the most rigid requirements for phytosanitary treatment, probit 9 mortality at 95% confidence level, because the minimum requirements required for the approved treatment schedules should be the upper limit in the confirmatory tests [48,51,53]. For the present MA-irradiation combined treatment of *T*. *granarium*, the treatment schedules can be described as a minimum exposure time of 15-d treatment under the maximum concentration of 1% O_2_ (with N_2_ balance) atmosphere after irradiation at the minimum absorbed dose of 200 Gy. For the phytosanitary application, both packaged (at normal atmosphere) or unpackaged grains and foodstuffs can be irradiated before export or at the port of entry, followed by packaging (including MA packaging, MAP) to prevent recontamination; and then the MA treatment may be conducted in the warehouse or during the transportation in sea container or train cabin or using MAP [15,17,33,54]. 

The synergistic coefficients (i.e., co-toxicity coefficient, synergistic ratios, synergistic factors) are typically used to evaluate the additive or synergistic effects of the joint action of insecticidal compositions, in most cases, it is a combination of two chemicals [46,55,56,57]; while the interaction effects (two-way or three-way ANOVA) have been analyzed to test synergistic effects for the combinations of chemical and physical conditions or multiple physical treatments, for example, ionizing radiation in combination with essential oil or cold storage [49,58]. In the present combined treatments, all the interaction effects among the treatment parameters (radiation dose, oxygen level, exposure time) were highly significant (Table 2, Figure 2), indicating that obvious synergistic effects present in all the MA-irradiation combinations. Moreover, the ANOVA may assist the determining of the optimum combination, the importance, and sequence of main factors, for example, we can only choose to apply radiation to modified MA and only take into account the LTs value of MA as a basis to calculate SRs (Formula (1)), because the main factor of radiation dose and interactions of dose × time were non-significant in the irradiation treatment alone (Figure 1). In addition, we also use the SRs to test the synergistic effects between MA and irradiation since there is no means to calculate the theoretical mortality induced by the two physical measures. As a result, all the SRs value was ˃1.47, especially, the mean values of SRs for late-stage larvae of *T*. *granarium* were 2.43 (1% O_2_) and 1.71 (2% O_2_) (Table 4). By the way, the SRs used in this study is more like the toxicity index used by Sun and Johnson (1960) for a pesticide mixture [55]. 

The biological effects of irradiation are to create damage to DNA that prevents multiplication and randomly inhibits cell functions, resulting in the death of the cell [22,33]. While the specific mechanisms by which insects are affected by and adapt to low-oxygen and high CO_2_ atmosphere remain poorly understood so far [23]. However, both irradiation and MA treatment can protect the treated food without the toxic residues left and cause a decrease in aerobic metabolism in insects, which may produce additive or synergistic effects (Table 3 and Table 4) to accelerate the death of insect pests [22,23,33]. Moreover, the efficacy of the two treatments on the different stages complements each other to provide a high level of quarantine security to regulated pests since the adult stage is more radiation tolerant but more sensitive to MA than the larval stages [3,5,28]. Although a longer exposure time (32.6-d at 24–26 ℃, Table 3) is needed to produce completely mortality of *T*. *granarium* under 1% O_2_ compared with the pure nitrogen (6-d at 30 ℃) or high CO_2_, but it is cheaper in use, more convenient to produce and implement in practice [3,17,59]; furthermore, a combined treatment time of 15-d at 24–26 ℃ (Table 5) may be acceptable, especially in the combination of phytosanitary treatment with international transportation [28,54].

Both ionizing radiation and MA treatment are ecofriendly phytosanitary measures alternative to methyl bromide fumigation in quarantine and pre-shipment (QPS) uses [16,22,59]. First of all, this combination has great significance on reducing the damage to stored grains and food staffs and long-term transportation of foodstuffs during international trade, because the khapra beetle is recognized as one of the most 100 invasive species that cause extremely high infestation levels to a wide range of stored products [1,9,60,61]. Secondly, *T*. *granarium* diapausing larvae were determined as the most tolerant to MA, but a 200 Gy irradiation could provide treatment efficacy at probit 9 to the most tolerant stage, the adults; therefore, another advantage for this combination is that the low-oxygen MA-irradiation combination could provide quarantine security at a high level, even if the diapausing larvae are presented in commodities [19,20,22]. Once again, elevated CO_2_ levels cause spiracles to open (remaining permanently open at ≥10% CO_2_) resulted in insect death from water loss and impact on the nervous system by its direct toxic effects. CO_2_ can also acidify the hemolymph leading to membrane failure in some cases [18,59]. Despite the similarities in response, arthropod mortality is generally greater in response to high carbon dioxide as exposure to the low-oxygen atmosphere [62]. Furthermore, when elevated CO_2_ is added to low-oxygen atmospheres, the additive or synergistic effects have been observed depending on the concentrations and the insect species used [62,63,64]. Therefore, a promising treatment may be established by combination low-dose irradiation (save cost and time) with a low-oxygen and high CO_2_ atmosphere to further shorten the exposure time (for MA treatment), and to be accepted by all the user (the exporter, importer, regulator) to an alternative to the QPS uses of methyl bromide.

MAP has been broadly used for controlling insect pests and maintain the quality of stored and perishable products. It is easy to realize the combination of MA, transportation, and storage together after irradiation treatment [17,33,34]. There is a potential for this treatment schedule to be used for the phytosanitary treatment of infested commodity on sea container, warehouse, MAP, or railway cabins, for example, the China-Europe Railway Express which takes two weeks or more [54]. A low-oxygen atmosphere may reduce the radiation effects [16,22,35,38,65]. The procedure for the application of MA-irradiation combination treatment should be conducting irradiation at normal atmosphere, firstly, followed by MA including the controlled atmosphere. However, mortality of *T*. *granarium* late-aged larvae decreased significantly with decreasing treatment temperature (35 °C ˃ 25 °C ˃ 0 °C under 1% low-oxygen atmosphere) [3,5,66]. Furthermore, other factors, such as insect stage and relative humidity, may affect the treatment efficacy, further research is still essential to compare tolerance difference in all the possible stages [22,29,48,59], to test the effect of temperature and radioprotection under a low-oxygen atmosphere, and evaluate the commodity quality under commercial conditions.

## 5. Conclusions

The combination treatment of low-oxygen MA and irradiation have been confirmed to be an effective measure to disinfest the khapra beetle that is highly resistant to each of the treatment, since a minimum of 32.6 (29.2–37.5) and 38.0 (35.1–41.7) days were required to achieve a mortality of 99.9968% at 95% confidence level for late-stage larvae (the most tolerant to each of the treatment) treated at 1% or 2% O_2_ atmosphere, respectively. A dose of 200 to 600 Gy radiation can be used to enhance the effect of MA treatment resulting in an obvious synergism even if the main effects of radiation dose and interaction effects of dose × time were insignificant in the radiation treatment alone. The interaction effects of two-way ANOVA, as well as the SRs, are used effectively to analyze the synergistic effects of the combination treatments; as a result, all the SRs are with 1.47 to 2.47, indicating that 32 to 60% of the exposure times are predicted to be saved comparing with MA alone. In addition, the probit estimation and synergistic effects were validated by treating a total of 111,366 late-stage larvae without survivors, and then the treatment schedules can be established for the phytosanitary disinfestation of the khapra beetles and other stored-product insects.

## Figures and Tables

**Table 1:** Exposure times for the dose-response tests on *T. granarium* larvae at 24–26 ℃.

Stage	X-ray	O_2_	Exposure Times (d)
middle-stage larvae	- -	1%	2, 4, 6, 8, 10, 12, 14, 16
		2%	2, 6, 10, 14, 18, 20
	200 Gy 200 Gy	1%	1, 2, 3, 4, 5, 6, 7
		2%	2, 4, 6, 8,10,12
late-stage larvae	- -	1%	2, 6, 10, 14, 18, 22, 24
		2%	4, 8, 12, 16, 20, 24, 28
	200 Gy 200 Gy	1%	2, 3, 4, 5, 6, 7, 8, 9, 10
		2%	2, 6, 10, 14, 18

**Table 2:** Mortality of *T. granarium* adult and larvae treated at 24–26 ℃ under 1% O_2_ atmosphere for the exposure times of 3, 6, and 9-d after 200, 400, and 600 Gy X-rays irradiation.

Stage	X-rays (Gy)	No. of Insects	Corrected Mortality (%) at Exposure Time of:				Stage Mortality (%)
			3-d	6-d	9-d	Mean ± SD	
Adults	200	1049	76.6 ± 1.3 cF	93.4 ± 3.6 bcE	100.0 ± 0.0 aD	90.0 ± 10.7 c	93.5 ± 8.4 a
	400	1049	83.5 ± 1.8 bF	98.2 ± 0.8 abE	100.0 ± 0.0 aD	94.0 ± 7.9 b	
	600	1016	90.0 ± 2.2 aF	99.7 ± 0.4 aE	100.0 ± 0.0 aD	96.6 ± 5.1 a	
Middle-stage larvae	200	1634	51.8 ± 2.2 cF	94.1 ± 0.5 bE	100.0 ± 0.0 aD	82.0 ± 22.8 c	87.3 ± 17.4 b
	400	1571	64.9 ± 0.7 bF	96.8 ± 0.5a bE	100.0 ± 0.0 aD	87.2 ± 16.8 b	
	600	1598	78.2 ± 1.7 aF	99.8 ± 0.2 aE	100.0 ± 0.0 aD	92.7 ± 10.9 a	
Late-stage larvae	200	1822	39.8 ± 0.4 cF	77.1 ± 1.3 cE	90.7 ± 0.4 cD	69.2 ± 22.8 c	77.4 ± 20.6 c
	400	1718	51.6 ± 0.6 bF	85.9 ± 1.3 bE	95.9 ± 0.7 bD	77.8 ± 20.1 b	
	600	1709	62.3 ± 1.4 aF	94.0 ± 0.9 aE	99.3 ± 0.5 aD	85.2 ± 17.4 a	

Within each column, means followed with different lowercase letters within a stage are significantly difference (*p* ˂ 0.05; Tukey test); means followed by different capital letters in the same row are significantly different (*p* < 0.05, Tukey test).

**Table 3:** Estimating the minimum lethal time for middle- and late-stage larvae of *T. granarium* treated at 24–26 ℃ under 1% or 2% O_2_ atmosphere alone or in combination with 200 Gy X-ray irradiation.

Treatment	Stages	No. Insects	Slope ± SE	Intercept ± SE	Estimated Lethal Time (95% CIs) (d) *			Hetero-geneity
					LT_90_	LT_99_	LT_99.9968_	
1%O_2_	middle-	1970	0.261 ± 0.012	−1.188 ± 0.080	9.5 (9.0–9.9) e	13.5 (12.7–14.4) e	19.9 (18.5–21.5) de	1.26
	late-	1829	0.168 ± 0.007	−1.474 ± 0.096	16.4 (15.5–17.5) b	22.6 (21.1–24.5) b	32.6 (29.2–37.5) b	2.21
2%O_2_	middle-	3492	0.281 ± 0.008	−1.953 ± 0.060	11.5 (11.0–12.1) c	15.2 (14.5–16.1) cd	21.2 (20.0–22.6) d	2.09
	late-	1948	0.152 ± 0.007	−1.774 ± 0.109	20.1 (19.4–20.9) a	27.0 (25.7–28.4) a	38.0 (35.1–41.7) a	1.30
1%O_2_ +200Gy	middle-	3550	0.583 ± 0.018	−1.516 ± 0.063	4.8 (4.6–5.1) h	6.6 (6.2–7.1) h	9.5 (8.8–10.3) h	3.26
	late-	2412	0.432 ± 0.014	−1.710 ± 0.074	6.9 (6.4–7.6) g	9.3 (8.6–10.4) g	13.2 (11.9–15.1) g	6.28
2%O_2_ +200Gy	middle-	2878	0.432 ± 0.014	−2.108 ± 0.076	7.8 (7.5–8.2) f	10.3 (9.8–10.8) f	14.1 (13.4–15.0) f	1.67
	late-	2333	0.222 ± 0.009	−1.180 ± 0.078	11.1 (10.1–12.4) cd	15.8 (14.2–18.2) c	23.4 (21.5–27.7) c	5.67

Within a column, estimated value followed by different letter are significantly difference (lethal dose ratio test at *p* ˂ 0.05).

**Table 4:** Synergistic ratios for the middle- and late-stage larvae of *T. granarium* treated at 24–26 ℃.

Treatment	Larval Stage	Synergistic Ratios Based on:		
		LT_90_	LT_99_	LT_99.9968_
1%O_2_ + 200Gy	middle-	1.98	2.05	2.09
	late-	2.38	2.43	2.47
2%O_2_ + 200Gy	middle-	1.47	1.48	1.50
	late-	1.81	1.71	1.62

**Table 5:** Results of the confirmatory tests on *T. granarium* late-stage larvae treated at 24–26 ℃ under 1% O_2_ atmosphere for 13~15 days after 200 Gy irradiation.

Date of Treatment	Treatment	No. of Insects	Exposure Time (d)	No. of Survivor
11 August 2020	X-rays	10,000	13	1
	X-rays	10,000	14	0
	X-rays	10,000	15	0
	control	3400	15	3325
5 September 2020	X-rays	25,374	15	0
	control	3600	15	3518
28 September 2020	X-rays	21,868	15	0
	control	2200	15	2130
21 October 2020	γ-rays	13,200	15	0
	control	1032	15	1012
31 October 2020	X-rays	19,904	15	0
	control	3100	15	3015
21 November 2020	X-rays	11,020	15	0
	control	2300	15	2240

The uncertainty for X-ray dose was 5%, and the monitored absorbed dose for gamma radiation was 173.9–199.8 Gy.

## Data Availability

All data presented in this study are available in the article.
